# Predictors and reasons for inappropriate hospitalization days for surgical patients in a tertiary hospital in Wuhan, China: a retrospective study

**DOI:** 10.1186/s12913-021-06845-y

**Published:** 2021-09-01

**Authors:** Hao Li, Hongbing Tao, Gang Li

**Affiliations:** 1 School of Medicine and Health Management, Tongji Medical College, Huazhong University of Science and Technology, 430030 Wuhan, China; 2 Department of Outpatient Management, Tongji Hospital, Tongji Medical College, Huazhong University of Science and Technology, 430030 Wuhan, China

**Keywords:** C-AEP, Delay tool, Inappropriate hospitalization day, Length of hospital stay

## Abstract

**Background:**

Inappropriate hospitalization day (IHD) is recognized as an important indication of the excessive demand for health-care services, especially for surgical patients. We aim to examine the degree of IHDs, predictors associated with higher incidences of IHDs, and reasons for each IHD in different periods of hospitalization.

**Methods:**

A total of 4586 hospital days from 408 cases were evaluated by a cross-sectional and retrospective audit program carried out in a tertiary hospital with 5613 beds and 9623 faculty in Wuhan, China. This study used the revised Chinese version of the Appropriateness Evaluation Protocol (C-AEP) to assess IHDs, and the Delay Tool to ascertain each reason for IHDs. A binary logistic regression model was performed to examine the predictors of higher incidences of IHDs.

**Results:**

The average frequency of IHDs was 23.24 %, and a total of 322 cases (78.92 %) were reported to have experienced at least one IHD. The multivariate analysis showed that patients at the age of 60–69 with respect to under 50, and with overlength of stay were predictors of higher incidences of preoperative IHDs, while admission from outpatient, multiple diagnosis, higher surgical incision level, and overlength of stay were predictors of higher incidence of postoperative IHDs. The most frequent reasons related to health providers for IHDs were doctor’s conservative views of patient management and delays in inspection, prescription, appointment, or result report. Patient factors gave rise to nearly a quarter of postoperative IHDs.

**Conclusions:**

Findings from this study indicate that measures including paying more attention to the construction of MDT for diagnosis and treatment in general surgery, reducing laboratory turnaround time, dispelling distrust among health-care providers and patients, setting stricter discharge standards and, providing integrated out-of-hospital services could be adopted accordingly to improve the inappropriateness of hospital stays.

**Supplementary Information:**

The online version contains supplementary material available at 10.1186/s12913-021-06845-y.

## Background

China has been targeted to amplify the utilization and accessibility of health services in recent years [1]. Between 2009 and 2020, the Government of China successively introduced policies focused on the expansion of health insurance and equities in health, meanwhile, impose restrictions on the excessive growth of medical expenses and supplier-induced demand in hospitals [2]. However, the regulations seemingly achieved little. As life expectancy continues to soar with the rapid acceleration of urbanization in China, the proportion of those over 65 scales up. These phenomena are associated with increases in the non-communicable disease (NCD) burden [3]. Furthermore, the total health expenditure of the Chinese mainland has dramatically reached 6519.59 billion CNY in 2019 partly on the account of the extensive waste of medical resources since the nationwide health care reforms in 2009, with an average growth rate of nearly 14.2 % over the past 10 years which is generally rising faster than overall economic growth [4].

As health expenditures continue to increase, policy-makers and hospital managers come to realize the significance of reducing excessive utilization of health-care services. The hospital system is by far regarded as the largest single component of health expenditure and the dominating driver of increased health-care spending in various countries [5]. In addition, unreasonable utilization of hospital services has been widely reported among health providers, including inappropriate admissions and prolonged hospital stays. Notably, there was a jump in the number of discharged patients by nearly double from 133 million to 270 million between 2009 and 2019 in China [4] due to the rapid increases in health insurance coverage [6]. In contrast, outpatient utilization increased only moderately. The number of hospital beds in China is relatively low compared with the Organization for Economic Co-operation and Development (OECD) data available, and at 4.3 per 1000 inhabitants [7]. However, the average length of stay (ALOS) in China is longer than the OECD averages. In 2019, the ALOS in public hospitals in China was 9.3 days, while in OECD countries, the average was 6.5 days [8]. This discrepancy further reflects the diversities in the clinical practice of different health-care system and the potential existence of inappropriate hospitalization days (IHDs).

IHD had been defined as a hospital stay that serves no clinical purpose and indicates inefficient resource utilization for hospital services that could have been provided in an uncomplicated health-care setting and at a lower cost [9, 10]. Current evidence suggests that inappropriate use of hospital services remains widespread [11–13]. Prolonged hospital stays implied the overall inefficiency of medical services [14], and besides, they were also found to be highly associated with the greater economic burden of disease and the increased risk of hospital-acquired infections [15–17], meanwhile, could further reduce the access of critically ill patients to inpatient services when medical resources were limited [18]. Therefore, the shrinkage of IHDs can optimize the process of services for a high-quality experience, and improve the efficiency of services delivery. More importantly, it has been identified as a highly effective way to reduce hospitalization expenses without compromising the quality of these services [19]. Whereas avoiding IHDs is undeniably difficult because the mechanisms that yield IHDs are multi-factorial [20]. While reform proposals have been debated vigorously, less attention has been paid to monitoring frameworks for hospitals and systematic evaluation [5]. In general, the number of IHDs can be reduced to a large extent through implementing quality improvement measures. However, it should be noted that the first step in every quality improvement process is the identification of the problem and its extent.

Utilization review (UR) is a cost-restriction project targeted to reduce medically unnecessary procedures by determining whether a health-care service is provided according to the appropriateness of intensity and cost [21]. Most studies that assessed the appropriateness of hospital services had been conducted in western countries, using the Appropriateness Evaluation Protocol (AEP) and the hospitalization days Delay analysis tool (Delay tool). AEP is an objective standard for evaluating the adequacy of acute-stage treatment admission and follow-up inpatient health services. The initial prototype based on clinical and technical standards was originally developed in 1981 by Gertman and Restuccia [9], and had been successively adapted for departments of internal medicine, pediatrics, gynecology, and surgery. In 1993, Austria, France, Italy, Portugal, Spain, Sweden, and the UK established a unified AEP standard for EU countries [22, 23]. In China, the Chinese version of AEP (C-AEP) [24] was recently introduced for assessing the appropriateness of admission and hospitalization in various levels of hospitals. In contrast, the Delay tool which focused more on the context of hospital stays for systematically evaluating reasons for IHDs was rarely used in China [25].

As for this complex problem, the identification of associated factors and specific reasons for IHDs is crucial for an understanding of this phenomenon. There are many studies about the degree of inappropriate use and determinants of IHDs. However, the published papers vary greatly in their methodology and the type of patient considered. For example, younger age, self-pay, outpatient admission, and inappropriate admission were found to be predictors of higher levels of IHDs in the cardiology department, while were not applicable for orthopedics department even in the same tertiary hospital [26]. In addition, certain indicators may exert the opposite influence on IHDs in different studies [27–29]. Few studies in China have focused merely on surgery settings, and as far as we know, no specific analysis was conducted according to concrete periods within the hospitalization episode. Therefore, in order to better understanding the circumstances of IHDs, we aimed to quantify the degree of IHDs in different periods of hospitalization and to analyze which factors are associated with higher incidences of IHDs based on revised C-AEP. Besides, we also proposed to ascertain the reasons for each IHD by Delay tool and to further provide evidence-based references to reduce IHDs.

.

## Methods

### Study setting

This study was a cross-sectional and retrospective audit program targeted to explore possible predictors of IHDs and to ascertain the reasons why patients with surgery had IHDs in a third-level teaching hospital with 5613 beds and 9623 faculty in Wuhan, China. There were several methods to measure the IHDs, and the AEP was the most commonly used among them. This study used the revised C-AEP to access the appropriateness of hospital stays, and the Delay Tool to ascertain each reason for IHDs. Conduction of this audit program had been performed in accordance with the Declaration of Helsinki and reported to the Tongji Medical School Ethics Commission. In addition, approval was obtained beforehand. An explanation of this study was given to patients and their family members. Those who agreed to participate with informed consent were enrolled in the audit program. Participant’s privacy and confidentiality were protected.

### Evaluation instrument

#### C-AEP

The assessment tool adopted in this study was adapted from the C-AEP developed by Liu W [24] in 2015. The original C-AEP was developed for both medical and surgical settings, and we found it was to some extent redundant for our study focusing merely on surgical patients. Therefore, the Delphi method was used to revise and simplify the C-AEP for highly specialized surgical settings. Compared with the original C-AEP, we had made specific adjustments, for example, replacing B3-B6 criteria (cardiac catheterization, angiography, thoracentesis or paracentesis, and invasive central nervous system diagnostic procedure that day) of the original C-AEP by “surgical or invasive operation on that day”. Furthermore, the inter-observer reliability and the convergent validity of the kappa values were 0.743, 0.691 for patient days, proving the revised C-AEP is a very sensitive and relatively specific tool with satisfactory predictive power to evaluate the IHDs in China. The final revised C-AEP in our study consists of three kinds of criteria: criteria relating to medical activity, criteria relating to nursing/life support services, and criteria relating to the condition of patients, with a total of 19 items. For our study, we defined a hospital stay as IHD if the patient had not met any of the 19 explicit criteria in the AEP standard of Appendix 1.

#### C-Delay Tool

The Chinese version of the delay tool for hospital days (C-Delay Tool) was used to ascertain reasons for IHDs [30]. The C-Delay tool in our study was initially developed based on the literature research and the Delphi method. Selker reviewed the medical records of the case and developed a tool for evaluating reasons for IHDs [25]. In general, the reasons for IHDs could be summarized into 9 main categories and 166 subcategories. The first step of tool adaptation in our study was to translate the original items into Chinese. A bilingual doctor of medicine was recruited to perform the translation from English to Chinese. Then, a clinical physician who had English-speaking background was appointed to conduct a backward translation for confirmation. Finally, divergences in translation were compared and discussed until they reached a consensus. Besides, to better adapt the Delay tool to China’s health-care settings, twenty-five experts engaged in clinical work over 10 years were successively invited to participate in two rounds of consultations. We had eventually retained 21 items that may trigger IHDs for patients in tertiary hospitals, involving factors related to medical providers, patients, and socioeconomic status classified through factor loading matrix as shown in Appendix 2.

### Data collection

#### Selection criteria

 According to the International Classification of Diseases, Ninth Revision, Clinical Modification (ICD-9-CM), we had calculated the top 50 volume of surgical procedures and eliminated the obstetrics, gynecology, thyroid, and breast surgery cases which indicated significant disequilibrium in gender distribution. In order to better present typical types of surgery in our study, three typical surgical procedures were elaborately selected, including “laparoscopic cholecystectomy”, “spine fusion implantation”, “thoracoscopic lobectomy”. And the ICD codes of these surgeries were 51.2, 84.5, and 32.4, respectively.

Patients who had been admitted from January 2014 to February 2015 with these corresponding ICD codes, and agreed to participate with informed consent were eligible for this study. Patients under 16-years-old, died or discharged over 30 days or with severe acute complications were excluded. In addition, patients whose medical records with inconsistent operation codes on the first page and length of hospital stays over 30 days were also ineligible since hospital managers would intervene to help reduce prolonged hospital stays from the 31st day of hospitalization which might undermine the authenticity of their inappropriateness. All records of patients with these corresponding ICD codes were extracted from the electronic medical records system and we had assessed 1314 cases for eligibility. According to the selection criteria, we had eventually got 408 participants who agreed to sign informed consent and to share their medical records for our audit program.

#### Outcome and variable of interest

Our primary outcome of interest was whether surgical patients had experienced IHDs and what extent of IHDs had they experienced. For each case, two well-trained medical staff from corresponding departments (a nurse and a clinician who had not been involved directly in the treatment process) simultaneously performed in-depth chart review to abstract relevant data. Each of them could fully comprehend the indications for each item and was required to identify the appropriateness of each hospital day one by one in order to discern the inappropriate one.

In addition to the appropriateness of hospital days, the following data were collected for each patients: patient socio-demographic information included age, gender (male vs. female), marital status (married vs. non-married, where non-married was defined as single, divorced, separated, or widowed), location of residence (Wuhan vs. others), and self pay (yes/no); patient condition included severity of illness (moderate vs. severe), multiple diagnosis (single, defined as a single diagnosis without complications and comorbidities; multiple, defined as patient with the presence of additional diagnosis); hospitalization-related factors included admission approach (outpatient vs. emergency), first hospitalization (yes/no), overlength of stay (yes/no, defined as the extremely prolonged hospital stays that exceed the average length of stay by a standard deviation); surgery-related factors included types of surgery, blood transfusion (yes/no), incision grade(0 and I vs. II or over), use of ventilator (yes/no), additional surgery (yes/no, defined as patients with more than one surgical operation).

Reasons for each IHD were ascertained and sorted out according to the Delay tool. Any questions or discrepancies would be addressed by an appointed supervisor to reach a consensus.

### Statistical analysis

From previous research, we had identified possible determinants including age, gender, marital status, location of residence, admission approach, severity of illness length of stay, disease category, complications, and the payment method [26–29, 31, 32]. In addition, we included some specific variables about surgical patients that we considered likely to be associated with IHDs.

In the univariate analysis, we summarized our data to present the distribution of IHDs as means and standard deviations for continuous variables, and as frequencies or percentages of patients for categorical variables. The t-test was used to assess the statistical significance of means differences by group for the level of IHDs. We used the chi-square test to identify significant differences in the frequencies of IHDs according to patient characteristics and utilization of medical services.

Multicollinearity for the regression analysis was checked by reviewing the values of variance inflation factor (VIF = 1/tolerance) or tolerance. In our study, types of surgery were excluded from the equation for the multicollinearity problem.

The binary logistics regression model was run, adopting whether experiencing IHDs or not as the dependent variable. All the following factors as explanatory variables (age, admission approach, multiple diagnosis, incision grade, use of ventilator, overlength of stay) with statistical significance based on the univariate analyses were entered into the equation.

Odds ratio (OR) are given with the corresponding 95 % confidence intervals (CI), and p levels. Statistical significance was set at p < 0.05. All data entries and analyses were performed using SPSS 24.0 software.

## Results

### Appropriateness assessment

A total of 4586 hospital days from 408 cases were reviewed, and 1066 (23.24 %) hospital days were found to be inappropriate, with 78.92 % of surgical patients determined to have had at least one IHD. We also found that there was a statistically significant difference in the frequency of IHDs between pre-operation and post-operation hospital stays (P < 0.001), and the conditions of patients were quite diverse as well [33]. Therefore, the IHDs were analyzed separately according to different phases of hospitalization. As the level of IHDs shown in Table 1, the percentage of inappropriate hospitalizations before and after surgery were accordingly 53.92 and 52.70 %. For the included patients, the average length of total stay was 11.24 days and the inappropriate of these was 2.62 days. The average pre-operation and post-operation hospital stays were 4.6 days and 6.64 days, respectively, with 1.19 days (25.80 %) and 1.43 days (21.48 %) for IHDs.

**Table 1:** The levels of IHDs in different phases of hospitalizations

Phases of hospitalizations	Total hospital days, n	Total IHDs, n (%)	LOS, m (SD)	IHDs, m (SD)	*p*
Pre-operation	1876	484 (25.80 %)	4.60 (2.39)	1.19 (1.48)	< 0.001
Post-operation	2710	582 (21.48 %)	6.64 (3.25)	1.43 (1.76)	
The whole hospitalization	4586	1066 (23.24 %)	11.24 (4.49)	2.62 (2.38)	

Notes: *m* mean; *SD* standard deviation; *IHDs* inappropriate hospitalization days; *LOS* length of stay

### Characteristics of patients with IHDs

Table 2 shows the characteristics and IHDs data for the subject patients. Patients in this study were middle-aged (mean 51.7 years), predominantly married (382/408, 93.6 %), admitted for the first time (367/408, 90.0 %), and covered by insurance (387/408; 94.9 %). In addition, most of them were admitted from the outpatient department (390/408, 95.6 %) and moderately ill (376/408, 92.2 %). Relatively few patients had experienced an overlength of hospital stays (70/408, 17.2 %).

**Table 2:** Characteristics of IHDs for surgical patients by different phases of hospitalization

Variable		Total	IHDs for whole hospitalization			IHDs before surgery			IHDs after surgery		
			Yes	None	*p*	Yes	None	*p*	Yes	None	*p*
		n(%)	n(%)			n(%)			n(%)		
Total	**Patients**		322	86		220	188		218	190	
Age (years)	**Under 50**	152 (37.3)	109 (33.9)	43 (50.0)	**0.031**	69 (31.4)	83 (44.1)	**0.018**	74 (33.9)	78 (41.1)	0.317
	**50–59**	145 (35.5)	117 (36.3)	28 (32.6)		81 (36.8)	64 (34.0)		77 (35.3)	68 (35.8)	
	**60–69**	91 (22.3)	79 (24.5)	12 (14.0)		60 (27.3)	31 (16.5)		55 (25.2)	36 (18.9)	
	**Over 69**	20 (4.9)	17 (5.3)	3 (3.5)		10 (4.5)	10 (5.3)		12 (5.5)	8 (4.2)	
Gender	**Male**	221 (54.2)	169 (52.5)	52 (60.5)	0.187	113 (51.4)	108 (57.4)	0.219	122 (56.0)	99 (52.1)	0.435
	**Female**	187 (45.8)	153 (47.5)	34 (39.5)		107 (48.6)	80 (42.6)		96 (44.0)	91 (47.9)	
Marital status	**Married**	382 (93.6)	302 (93.8)	80 (93.0)	0.796	209 (95.0)	173 (92.0)	0.22	204 (93.6)	178 (93.7)	0.965
	**Non-married**	26 (6.4)	20 (6.2)	6 (7.0)		11 (5.0)	15 (8.0)		14 (6.4)	12 (6.3)	
Location of residence	**Wuhan**	118 (28.9)	92 (28.6)	26 (30.2)	0.763	61 (27.7)	57 (30.3)	0.565	62 (28.4)	56 (29.5)	0.818
	**Others**	290 (71.1)	230 (71.4)	60 (69.8)		159 (72.3)	131 (69.7)		156 (71.6)	134 (70.5)	
Self pay	**Yes**	21 (5.1)	16 (5.0)	5 (5.8)	0.753	8 (3.6)	13 (6.9)	0.135	11 (5.0)	10 (5.3)	0.921
	**No**	387 (94.9)	306 (95.0)	81 (94.2)		212 (96.4)	175 (93.1)		207 (95.0)	180 (94.7)	
Severity of illness	**Severe**	32 (7.8)	22 (6.8)	10 (11.6)	0.142	16 (7.3)	16 (8.5)	0.643	12 (5.5)	20 (10.5)	0.060
	**Moderate**	376 (92.2)	300 (93.2)	76 (88.4)		204 (92.7)	172 (91.5)		206 (94.5)	170 (89.5)	
Multiple diagnosis	**Single**	220 (53.9)	169 (52.5)	51 (59.3)	0.260	122 (55.5)	98 (52.1)	0.502	105 (48.2)	115 (60.5)	**0.012**
	**Multiple**	188 (46.1)	153 (47.5)	35 (40.7)		98 (44.5)	90 (47.9)		113 (51.8)	75 (39.5)	
First hospitalization	**Yes**	367 (90.0)	293 (91.0)	74 (86.0)	0.175	198 (90.0)	169 (89.9)	0.972	199 (91.3)	168 (88.4)	0.337
	**No**	41 (10.0)	29 (9.0)	12 (14.0)		22 (10.0)	19 (10.1)		19 (8.7)	22 (11.6)	
Admission approach	**Outpatient**	390 (95.6)	313 (97.2)	77 (89.5)	**0.002**	212 (96.4)	178 (94.7)	0.409	215 (98.6)	175 (92.1)	**0.001**
	**Emergency**	18 (4.4)	9 (2.8)	9 (10.5)		8 (3.6)	10 (5.3)		3 (1.4)	15 (7.9)	
First hospitalization	**Yes**	367 (90.0)	293 (91.0)	74 (86.0)	0.175	198 (90.0)	169 (89.9)	0.972	199 (91.3)	168 (88.4)	0.337
	**No**	41 (10.0)	29 (9.0)	12 (14.0)		22 (10.0)	19 (10.1)		19 (8.7)	22 (11.6)	
Overlength of stay	**Yes**	70 (17.2)	68 (21.1)	2 (2.3)	**0.001**	76 (34.5)	7 (3.7)	**0.000**	73 (33.5)	14 (7.4)	**0.000**
	**No**	338 (82.8)	254 (78.9)	84 (97.7)		144 (65.5)	181 (96.3)		145 (66.5)	176 (92.6)	
Types of surgery	**LC**	126 (30.9)	91 (28.3)	35 (40.7)	**0.000**	62 (28.2)	64 (34.0)	0.260	58 (26.6)	68 (35.8)	**0.000**
	**SFI**	144 (35.3)	100 (31.1)	44 (51.2)		85 (38.6)	59 (31.4)		39 (17.9)	105 (55.3)	
	**TL**	138 (33.8)	131 (40.7)	7 (8.1)		73 (33.2)	65 (34.6)		121 (55.5)	17 (8.9)	
Blood transfusion	**Yes**	4 (1.0)	3 (0.9)	1 (1.2)	0.847	2 (0.9)	2 (1.1)	0.874	3 (1.4)	1 (0.5)	0.985
	**No**	404 (99.0)	319 (99.1)	85 (21.0)		218 (99.1)	186 (98.9)		215 (98.6)	189 (99.5)	
Incision grade	**0 and I**	138 (33.8)	101 (31.4)	37 (43.0)	**0.042**	77 (35.0)	61 (32.4)	0.587	53 (24.3)	85 (44.7)	**0.000**
	**II or over**	270 (66.2)	221 (68.6)	49 (57.0)		143 (65.0)	127 (67.6)		165 (75.7)	105 (55.3)	
Use of ventilator	**Yes**	54 (13.2)	47 (14.6)	7 (8.1)	0.116	31 (14.1)	23 (12.2)	0.581	38 (17.4)	16 (8.4)	**0.007**
	**No**	354 (86.8)	275 (85.4)	79 (91.9)		189 (85.9)	165 (87.8)		180 (82.6)	174 (91.6)	
Additional surgery	**Yes**	241 (59.1)	191 (59.3)	50 (58.1)	0.844	138 (62.7)	103 (54.8)	0.104	121 (55.5)	120 (63.2)	0.117
	**No**	167 (40.9)	131 (40.7)	36 (41.9)		82 (37.3)	85 (45.2)		97 (44.5)	70 (36.8)	

Notes: *LC* laparoscopic cholecystectomy; *SFI* spine fusion implantation; *TL* thoracoscopic lobectomy

A total of 322 cases were found to experience at least one IHD during the whole hospitalization. Interestingly, patients with overlength of stay were found to be a strong indicator for IHDs (P < 0.001), regardless of different phases of hospitalization.

Patients with different age groups were found to have different incidences of IHDs during the pre-operation hospital stays (P = 0.018), while no significant difference was observed after surgery (P = 0.317).

In contrast, according to the result presented in Table 2, there was no difference for patients with different surgeries in terms of preoperative IHDs (P = 0.260), while patients were reported to have a statistically different incidence of IHDs during the post-operation hospital stays (P < 0.001). Besides, patients with multiple diagnosis (P = 0.012) or admitted from outpatient (P = 0.001) were more likely to experience IHDs during the post-operation hospital stays. No statistical difference among these groups was found in terms of gender, marital status, location of residence, whether the patients paid the bills by themselves, severity of illness, first hospitalization, blood transfusion, and additional surgery.

### The predictors for IHDs

The regression equation was statistically significant. As shown in Table 3, it turned out that age was partly significant in predicting IHDs at the preoperative stage. Surgical patients in the age group 60–69 are 2.167 times (OR = 2.167, 95 %CI = 1.217–3.862) more likely to experience IHDs compared with the patients who are aged under 50. However, there was no significant difference among other age groups.

**Table 3:** Results of binary logistic regression model of predictors for IHDs

Variable	β Coefficient	SE	OR	95 % CI	*p*
**IHDs before surgery**					
Constant	-0.522	0.179	0.593	-	0.003
Age (reference: under 50 years)					
50–59 years	0.378	0.252	1.459	[0.891, 2.391]	0.134
60–69 years	0.774	0.295	2.167	[1.217, 3.862]	0.009
Over 69 years	-0.143	0.544	0.866	[0.298, 2.517]	0.792
Overlength of stay	2.609	0.414	13.584	[6.039, 30.556]	0.000
**IHDs after surgery**					
Constant	-2.673	0.691	0.069	-	0.000
Use of ventilators	0.488	0.353	1.629	[0.815, 3.257]	0.167
Admission from outpatient	1.843	0.679	6.315	[1.669, 23.897]	0.007
Multiple diagnosis	0.501	0.221	1.65	[1.07, 2.544]	0.023
Higher surgical incision level	0.696	0.226	2.007	[1.288, 3.127]	0.002
Overlength of stay	1.805	0.329	6.08	[3.191, 11.585]	0.000
**IHDs for whole hospitalization**					
Constant	-1.01	0.577	0.364	-	0.08
Age (reference: under 50 years)					
50–59 years	0.502	0.294	1.653	[0.929, 2.94]	0.087
60–69 years	0.932	0.372	2.541	[1.226, 5.264]	0.012
Over 69 years	0.693	0.667	2	[0.541, 7.386]	0.299
Admission from outpatient	1.603	0.56	5.142	[1.421, 18.614]	0.004
Higher surgical incision level	0.391	0.262	1.479	[0.884, 2.472]	0.136
Overlength of stay	2.402	0.741	11.041	[2.586, 47.138]	0.001

Notes: *SE* Standard error; *OR* Odds ratio; *CI* Confidence interval

At the postoperative stage, use of ventilators (OR = 1.629, 95 %CI = 0.815–3.257) was not statistically significant in predicting IHDs, while patients who had multiple diagnosis (OR = 1.65, 95 %CI = 1.07–2.544), higher surgical incision levels (OR = 2.007, 95 %CI = 1.288–3.127), and been admitted from outpatient department (OR = 6.315, 95 %CI = 1.669–23.897) were more likely to experience IHDs. Furthermore, patients with overlength of stays were positively associated with IHDs during the whole hospital stays (*P* < 0.001).

### The reasons for IHDs

The results of the specific reason analysis were shown in Table 4. The reasons for IHDs mainly included medical factors and patient factors. Overwhelming superiority (86.99 %) of IHDs generated as a result of medical factors. The reasons for IHDs were relatively concentrated, the top five reasons accounted for more than 90 % of all IHDs, which were “Doctor’s conservative views of patients management” (39.40 %), “Delays in inspection, prescription, appointment or result report” (21.86 %), “Request by patient or family member for extended stay” (12.66 %), “Delays in operation” (11.07 %), “Delays due to lack of operating rooms or tables which hinders the punctuality of the operation” (9.01 %).

**Table 4:** Rate of each reasons explaining IHDs by different phases of hospitalization

Reasons			Whole hospitalization	Pre-operation	Post-operation
			n (%)		
Medical factors	A9	Doctor’s conservative views of patient management	425 (39.40 %)	8 (1.65 %)	417 (71.65 %)
	A2	Delays in inspection, prescription, appointment or report	233 (21.86 %)	211 (43.60 %)	22 (3.78 %)
	A4	Delays in operation (including preoperative waiting, inadequate surgical preparation, etc.)	118 (11.07 %)	116 (23.97 %)	2 (0.34 %)
	A7	Delays due to lack of operating rooms or tables which hinders the punctuality of the operation	96 (9.01 %)	94 (19.42 %)	2 (0.34 %)
	A3	Delays in expert consultation	26 (2.44 %)	26 (5.37 %)	/
	A10	Waiting for bed arrangement when patients are transferred to hospital	17 (1.59 %)	14 (2.89 %)	3 (0.51 %)
	A12	Patients were not able to determine treatment options or obtain informed consent	13 (1.2 %)	13 (2.68 %)	/
	A11	Over-examination or over-treatment in hospital	2 (0.19 %)	/	2 (0.34 %)
	A1	Shortages of medical staff in hospital	1 (0.09 %)	1 (0.21 %)	/
Patient factors	B1	Request by patient or family member for prolonged stay	135 (12.66 %)	3 (0.62 %)	132 (22.68 %)
	B2	The patient refused to discharge or transfer	2 (0.19 %)	/	2 (0.34 %)
Total			1066 (100 %)	484 (100 %)	582 (100 %)

The reasons for IHDs in different stages of hospitalization were quite diverse. As shown in Table 4, medical factors led to more than 99 % of IHDs for surgical patients during the pre-operation hospital stays. The top three factors were “Delays in inspection, prescription, appointment or result report” (43.60 %), “Delays in operation” (23.97 %), and “Delays due to lack of operating rooms or tables which hinders the punctuality of the operation” (19.42 %). At the postoperative stage, the gap between medical factors and patient factors had narrowed remarkably (respectively 76.98 and 23.02 %). The top three factors accounted for the most were “Doctor’s conservative views of patients management” (71.65 %), “Request by patient or family member for extended stay” (22.68 %), and “Delays in inspection, prescription, appointment or result report” (3.78 %).

## Discussion

This study indicated that IHDs occurred in approximately 23.31 % of total hospital days among the surgical patients from three typical departments of this tertiary hospital. Accordingly, the finding in our study was similar to those of a tertiary hospital in Beijing (20.76 %) [34], and a cardiology department in Shanghai (25.2 %) [26]. However, it was relatively higher compared with other studies (6.3 %~16.6 %) [35] in China. These discrepancies in the frequency of IHDs were firmly related to the fact that these studies had roughly used the original version of AEP for evaluation without adaption for certain contexts. Numerous published studies had demonstrated that the frequency of IHDs varied considerably in countries, hospitals, and clinical departments. Although the results of this study were consistent with Teke [31] using the Turkish version of AEP to review the evaluation results of 375 surgical cases in a military hospital in Turkey (21.3 %), and Fontaine’s [13] study on 10,921 hospital days in 23 hospitals in Belgium (24.61 %). Nevertheless, these comparisons in frequency could merely illustrate the extensive existence of IHDs, and hospitals even with the same level or volume could actually display various proportions of IHDs in different countries. For instance, Tavakoli (39.4 %) [32], Sangha (28 %) [36], Gautier (7 %) [37], Meidani (6.3 %) [38]. Therefore, it seemed necessary to introduce the adapted AEP tool to further comprehend the frequency of IHDs in certain contexts.

In addition, we found that nearly 80 % of surgical patients in our study had experienced at least one IHD, suggesting that the incidences of IHDs were even more widespread among surgical patients than previous studies revealed [39]. As previously mentioned, the relatively high incidences of IHDs may explain the underlying reason for longer ALOS in China. Hence, more attention should be paid to reduce IHDs since prolonged hospital stays would inevitably increase the financial burden of patients and the risks of hospital-acquired conditions [17, 18].

To the best of our knowledge, none of the already published studies had ever compared the IHDs and their determinants at different stages of hospitalizations (pre-operation vs. post-operation). Our findings suggested that there existed a significant difference in the proportions of IHDs (25.98 % verse 20.93 %). And patients in our study were more likely to experience IHDs during the pre-operation hospital stays, implying that there was still a lot of room for the shrinkage of preoperative IHDs.

Age had been identified as a predictor for IHDs in previous studies while the conclusions were multifarious. Studies conducted in China had demonstrated that older age was not a predictor for IHDs [26, 34]. In contrast, our findings indicated that the older age could partly predict higher incidences of preoperative IHDs which was in line with some previous studies [20, 40, 41]. To be specific, surgical patients at the age of 60–69 years were prone to experience IHDs compared with those who were under 50. This could be explained by the fact that older patients, even if their hospitalization partly did not meet the AEP criteria, may need other assistive care services [39]. However, there still existed a lack of alternative care services or caregivers at home in China, and older patients were therefore more likely to experience IHDs. Meanwhile, another possible reason for this was the clinical stereotypes that patients over 60 years were generally regarded as vulnerable groups who need more hospital stays for preoperative preparation, whereas surgical patients at the age of 60–69 actually had more stable health conditions than health-care providers thought. Interestingly, our study further revealed that apart from surgical patients at the age of 60–69 years, preoperative IHDs seemed to exist systematically among surgical patients, regardless of their characteristics. Besides, consistent with most previous studies [39, 42], our findings suggested that patients with the overlength of hospital stays were prone to experience IHDs. It seems reasonable that the more hospital days surgical patients had the more chances of IHDs they actually got.

At the postoperative stage, the presence of co-occurring diseases was positively linked with a higher incidence of IHDs. To some extent, patients with complications and comorbidities in this super-sized hospital usually needed the active involvement of the multi-disciplinary team (MDT), and whereas the clinical consultation would inevitably take few hospital days under the incomplete and inefficient MDT system in China [43, 44]. In addition, patients with a higher grade of incision, which was positively associated with surgical incision infections [45], were more likely to have IHDs. This finding further indicated that medical providers’ fear of postoperative complications may impose restrictions on the approval of discharge. Consistent with previous studies, we found the admission approach was a strong predictor for a higher incidence of IHDs, especially during the post-operation hospital stays. However, our finding indicated that patients admitted via the outpatient department for scheduled surgery were more likely to experience IHDs which contradicted the conclusion of a previous study conducted in Italy [29]. This discrepancy can be explained by the fact that the hospital in our study is the top ten teaching hospitals with great reputations in China, and consequently, thousands of patients with complex diseases are admitted each year while the previous study in Italy, in contrast, was conducted in the context of a hospital without highly-specialized services. Interestingly, this finding was not unexpected regarding to this tertiary hospital. To be specific, patients admitted for scheduled surgery had more stable and predictable functional conditions, and whereas surgical patients admitted via urgent referral or emergency department were more likely in severe clinical conditions, and badly in need of highly specialized services. This finding further revealed that predictors for higher incidences of IHDs might differ in their specific effects on hospitals at different levels. And further studies from different health-care systems or hospitals are urgently needed to better understand this phenomenon.

Factors related to medical providers were main triggers for IHDs, which was consistent with previous studies conducted by different researchers [46, 47]. It seems that researchers had already reached a consensus that health-care providers played dominant roles in manipulating IHDs and inducing unnecessary demand of hospital services [48]. Hence, measures were urgently needed for hospital managers or health-care providers to reduce IHDs. In our study, we found that the reason in relation to “Doctor’s conservative views of patients management”, which was less mentioned in previous studies, accounted for nearly half the factors related to medical providers. Health-care providers in China tended to manage patients according to the past clinical experience and go through in regular sequence without taking the patients’ specific conditions into consideration. This tendency might be associated with the “defensive” medicine in China triggered by the recently increasing tension and disharmony in doctor-patient relationships [49]. Furthermore, this could partly explain the results shown in Table 3 why the elderly patients were more likely to experience IHDs. High levels of distrust in providers were reported to be strongly associated with increased hospital utilization [50]. Under this scenario, elderly patients were deemed to be more vulnerable, and in need of observations by their family members. As a result, health-care providers knowingly over-treat the patients for sake of avoiding potential conflict.

“Delays in inspection, prescription, appointment or report” were another significant contributor to IHDs which was highly supported by previous studies [13]. Evidence showed that reducing laboratory turnaround time and improving the accuracy of diagnostic findings could shorten the ALOS and save hospital budget [51]. Meanwhile, this finding can illustrate why the surgical patients admitted through the outpatient department were prone to experience IHDs in our study since they were usually admitted for scheduled surgery in a selecting time which meant the patients’ conditions were stable to endure slight delays in preoperative hospital stays. In response to this phenomenon, we firmly believed that measures targeted to eliminate the lag of test results and improve the accuracy of the diagnosis were of great significance.

Factors related to patients were not supposed to be reckoned with. The reason regarding to “Request by patient or family member for extended stay” had reflected the excessive demands of hospital utilization due to the full coverage of basic medical insurance and the relevant reimbursement process in China [1]. Moreover, it was also connected with China’s hospital-centered and fragmented health-care delivery system [52]. Lacking a solid and integrated referral system, the continuity of service could not be guaranteed. And distrust from patients in primary care providers’ professional competence could be a barrier to ask for downward referrals. Challenges in terms of the primary health-care system’s structural weakness and financing policies further diminish its preparedness for the elderly population with the growing prevalence of NCDs. In this respect, stricter discharge standards and integrated out-of-hospital services were, to some degree, beneficial to reduce this part of the IHDs.

Our findings indicated that reducing IHDs should not merely rely on the one-sided effort from hospitals, and interventions should be realigned to take all stakeholders as a whole. Proactive discharge planning, detailed criteria for hospital stays, and increasing access to long-term care services were urgently needed to reduce potential IHDs.

### Limitation

This study has several limitations. First, it was conducted only in a tertiary hospital with highly specialized services, and the sampling of surgical patients was limited to only three categories. This could affect the generalizability of our results. Further studies conducted in hospitals at different levels and with more categories of surgical patients involved are needed to draw more general conclusions. Second, the audit program had not included the appropriateness of hospital admission due to the discontinuity of medical records, while previous studies indicated that inappropriate hospital admission was positively associated with higher incidences of IHDs [27, 28, 53]. Third, the application of AEP is regardless of categories of disease, however, different categories of disease might affect the distributions of reasons for IHDs. In addition, the reviewers of our audit program might spontaneously be subjective during the process even though they were initially well-trained. Artificial intelligence technology, like machine learning, can be applied to help eliminate the influence of contrived factors and reduce human costs in future studies.

## Conclusion

Findings from this study confirm that the frequency of IHDs was 23.24 % in this tertiary hospital in Wuhan, and additionally indicate that the characteristics of surgical patients from different phases of hospitalization should be considered to develop efficient and feasible interventions for IHDs reduction. The main causes of IHDs were medical factors that were related to the health-care providers, however, the patient factors gave rise to nearly a quarter of IHDs after surgery. These results indicate that measures including paying more attention to the construction of MDT for diagnosis and treatment in general surgery, reducing laboratory turnaround time, dispelling distrust among health-care providers and patients, setting stricter discharge standards and providing integrated out-of-hospital services could be adopted accordingly to improve the inappropriateness of hospital stays.

## Supplementary Information

**Additional file 1:**

## Data Availability

The datasets used and/or analyzed during the current study available from the corresponding author on reasonable request.

## Abbreviations

NCD: Non-communicable Disease
CNY: Chinese yuan
OECD: The Organization for Economic Co-operation and Development
ALOS: Average Length of Stay
IHD: Inappropriate Hospitalization Day
AEP: Appropriateness Evaluation Protocol
EMR: Electronic Medical Record
ICD: The International Classification of Diseases
LC: Laparoscopic Cholecystectomy
SFI: Spine Fusion Implantation
LL: Laparoscopic lobectomy
OR: Odds ratio
CI: Confidence interval
MDT: Multi-disciplinary Team
