# Supplementary material for: Predictors and reasons for inappropriate hospitalization days for surgical patients in a tertiary hospital in Wuhan, China: a retrospective study
Source: BMC Health Serv Res. 2021 Sep 1;21:900. doi: 10.1186/s12913-021-06845-y (PMC8408966; doi:10.1186/s12913-021-06845-y)
Supplement: Supplementary file 1 — Additional file 1: [file 12913_2021_6845_MOESM1_ESM.docx]

****Supplementary File****

**Appendix 1 The Revised C-AEP for Surgical Settings in Tertiary Hospital**

| **Health services (A)** | **Care/life support services (B)** | **Patient status (C)** |
| --- | --- | --- |
| A1 Surgical or invasive operation on the day | B1 Intermittent or continuous use of ventilator and/or inhalation treatment on the day | C1 Non-neural disorders or intestinal peristalsis in the past 24 hours |
| A2 Preoperative discussion and adequate preoperative preparation are needed on the day | B2 Intermittent or persistent intravenous nutritional support | C2 Ventricular fibrillation, acute ischemia, sinoatrial node dysfunction, atrioventricular block were recorded on the progress notes or electrocardiogram reports |
| A3 The dosage of new treatment or trial should be adjusted frequently according to patients’ conditions | B3 Continuous monitoring of vital signs is required on the day (At least once every 30 minutes for at least 4 hours) | C3 The axillary temperature >38℃ |
| A4 Patients had surgeries of level three or four in the last 24 hours | B4 Nursing of incision and drainage during major operation day | C4 Loss of consciousness(At least 1 hour) |
| A5 Multidisciplinary consultations are required on the day to develop treatment plans | B5 Being treated in ICU/CCU on the day | C5 Acute hematopathy, severe neutropenia, anemia, thrombocytopenia, leukocytosis, erythrocytosis, or thrombocytopenia with signs or symptoms |
|  |  | C6 The vital signs were abnormal <50 beats/min or >140 beats/min; systolic blood pressure <90 mm Hg or >200 diastolic blood pressure <60 mm Hg or 120 mm Hg. |
|  |  | C7 Severe electrolyte imbalance or abnormal blood gas or tested with the result of critical value |
|  |  | C8 Progressive or acute failure of organ or circulatory/dyspnea/ progressive acute nervous system lesions |
|  |  | C9 Exhiscence or active bleeding of the wound or blood transfusion due to bleeding |

**Appendix 2 Judgement tool of reasons for IHDs in Tertiary Hospital**

| **Factors related to medical providers (A)** | **Factors related to patients(B)** |
| --- | --- |
| A1 Inadequate of hospital medical staff | B1 Request by patient or family member for prolonged stay |
| A2 Delays in inspection, prescription, appointment or report. | B2 The patient refused to discharge or transfer |
| A3 Delays in expert consultation | B3 Patients with no identity, no responsible institutions/ personnel, or no treatment bugdet |
| A4 Delays in operation (including pre-operative waiting, inadequate surgical preparation, etc.) | B4 Inadequate out-of-hospital services or arrangements during transfers(e.g. insufficient beds in rehabilitation or care centres) |
| A5 Doctors suspend operations for personal reasons | B5 Communities can not provide skilled care services or home care services to patients |
| A6 Delays due to the evaluation of preoperative anesthesia which is not enough to determine the effective method of anesthesia | B6 Delays due to the waiting for referral |
| A7 Delays due to lack of operating rooms or tables which hinders the punctuality of the operation |  |
| A8 Delays due to the poor communication between medical staff or department |  |
| A9 Doctor's conservative views of patient management |  |
| A10 Waiting for bed arrangement when patients are transferred to hospital |  |
| A11 Over-examination, over-treatment |  |
| A12 Patients were not able to determine treatment options or sign informed consent |  |
